# Associations of dietary patterns with common infections and antibiotic use among Finnish preschoolers

**DOI:** 10.29219/fnr.v67.8997

**Published:** 2023-06-14

**Authors:** Henna Peltonen, Maijaliisa Erkkola, Anna M. Abdollahi, Marja H. Leppänen, Eva Roos, Nina Sajaniemi, Anne-Maria Pajari, Henna Vepsäläinen

**Affiliations:** 1 Department of Food and Nutrition, University of Helsinki, Helsinki, Finland; 2 Folkhälsan Research Center, Helsinki, Finland; 3 Faculty of Medicine, University of Helsinki, Helsinki, Finland; 4 Department of Food Studies, Nutrition and Dietetics, Uppsala University, Uppsala, Sweden; 5 Philosophical Faculty, School of Applied Educational Science and Teacher Education, University of Eastern Finland, Joensuu, Finland

**Keywords:** Flu, Stomach virus, Dietary habits, Day care centre, Kindergarten, Childcare centre

## Abstract

**Background:**

Preschoolers suffer frequently from infections. Although nutrition plays a key role in immune function, very little is known about the impact of overall diet on preschoolers’ infections.

**Objective:**

To assess the associations between dietary patterns, common infections and antibiotic use among Finnish preschoolers.

**Design:**

The study included 721 3–6-year-old preschoolers participating in the cross-sectional DAGIS survey. Parents retrospectively reported the number of common colds, gastroenteritis episodes and antibiotic courses their children had acquired during the past year. Food consumption outside preschool hours was recorded using a food frequency questionnaire. Dietary patterns were derived from the consumption frequencies using principal component analysis. Associations between the thirds of the dietary pattern scores and the outcomes were analysed using logistic and negative binomial regression models.

**Results:**

Prevalence of common colds was lower in moderate and high adherence to the sweets-and-treats pattern than in low adherence (prevalence ratio [PR]: 0.89, 95% confidence interval [CI]: 0.80–1.00, and PR: 0.88, 95% CI: 0.79–0.99, respectively) and higher in high adherence to the health-conscious pattern than in low adherence (PR: 1.13, 95% CI: 1.01–1.27) after adjusting for age, sex, number of children living in the same household, frequency of preschool attendance, family’s highest education and probiotic use. The risk of ≥1 gastroenteritis episode and the prevalence of antibiotic courses were lower in moderate adherence to the sweets-and-treats pattern than in low adherence (odds ratio [OR]: 0.63, 95% CI: 0.44–0.92 and PR: 0.77, 95% CI: 0.59–1.00, respectively).

**Conclusions:**

The results were unexpected. Parents who were most health-conscious of their children’s diet might also have been more aware of their children’s illness.

## Popular scientific summary

- We analysed cross-sectional associations between dietary patterns, common infections and antibiotic courses in Finnish preschoolers.
- Based on parents’ reports, a healthier dietary pattern was positively associated with the prevalence of common colds. An unhealthier dietary pattern was inversely associated with the prevalence of common colds and antibiotic courses, as well as the risk of gastroenteritis.
- Our unexpected results suggest that longitudinal designs and objective measures for assessing infections should be considered when investigating the subject.

Children attending preschool are at higher risk of contracting infections than children cared for at home (1–4). Upper respiratory tract and gastrointestinal infections are common and frequently experienced by preschoolers (5), although the common cold appears to be the most prevalent and recurrently encountered illness (5, 6). In addition to discomfort caused by infectious conditions, preschool-attributable illnesses often lead to parental absence from work, deficient utilisation of preschool, visits to physicians and prescriptions of antibiotics (2, 7). In the Netherlands, societal costs for care and treatment of gastroenteritis and influenza-like illnesses experienced by children attending preschool were estimated to be twice as high as those experienced by children not attending preschool (4). A similar trend may exist in other Western countries, where most young children attend preschool (8). It is therefore important to identify risk factors related to infections in preschoolers.

Nutrition has the potential to modify preschoolers’ predisposition to infections. Appropriate immune defence relies on sufficient intakes of energy (9) as well as numerous nutrients, such as protein (9), polyunsaturated fat (10), fibre (11), vitamin A (12), vitamin D (12), vitamin C (13), zinc (14) and iron (15), and their deficiencies can lead to a predisposition for recurrent infections (12, 16). However, diets high in sugar and saturated fat are common among children in Western countries (17), and these diets may interfere with mucosal immune defence (18–20). It is also known from previous research that the consumption of dairy containing probiotics or lactoferrin may prevent preschoolers’ infections (21–25), but nonsignificant results have also been reported (26–28). However, nutrients share complex interactions in immune function (29), and each food item contains multiple nutrients. As a diet is usually characterised by a mixture of different foods, it may not be meaningful to look at the separate effects of nutrients and food items (30, 31). Instead, a whole-diet approach such as dietary pattern analysis may be the most predictive in investigating the association between nutrition and infections among preschoolers (30–32). To the best of our knowledge, only one study to date has examined the importance of the whole diet for acquiring infectious diseases in preschoolers. In this study among Finnish preschoolers, a dietary pattern emphasising fruit and berry consumption was inversely associated with the prevalence of acute otitis media, while a dietary pattern emphasising biscuit and sweet pastry consumption was linked to higher odds of pneumococcal carriage (33).

To address the knowledge gap, we aimed to investigate whether data-derived dietary patterns are associated with common infections, namely common colds and gastroenteritis, among Finnish preschoolers using a validated food frequency questionnaire (FFQ) designed to assess the children’s diet as a whole. We also examined whether dietary patterns are associated with antibiotic use. We hypothesised that a healthier dietary pattern would be inversely associated with the infectious outcomes while an unhealthier dietary pattern would have a positive association.

## Materials and methods

### Study design and participants

This study was part of the Increased Health and Wellbeing in Preschools (DAGIS) research project. The overall aim of the project was to diminish socioeconomic differences in preschoolers’ energy balance-related behaviours. The study sample and the protocol have previously been described in detail (34, 35). Briefly, the cross-sectional survey was conducted between September 2015 and April 2016 in eight municipalities located in Southern and Western Finland. Families having 3–6-year-old children were recruited via contacting municipal or private preschools from whom the municipalities purchased education services. Eligible preschools had ≥1 group of children aged 3–6 years, were Finnish- or Swedish-speaking and provided day care only during the daytime. Of the contacted preschools, 86 (56%) were eligible and consented. Of the consenting preschools, all children in the age group 3–6 years (*N* = 3,592) and their families were invited to the survey. Altogether 864 children (24% of those invited) and their families from 66 preschools (43% of those invited) consented to participate, provided some data and formed the final survey sample. The study was conducted in accordance with the Declaration of Helsinki and was approved by the University of Helsinki Ethical Review Board in the Humanities and Social and Behavioural Sciences in February 2015 (Statement 6/2015).

### Dietary assessment

Children’s food consumption was measured using a nonquantitative FFQ designed to estimate the usual frequency of consumption. The FFQ included 47 food items from the following seven food groups: vegetables, fruits and berries; dairy products; fish; meat and eggs; cereal products; drinks; and others (i.e., sweets and snacks). The parents reported how many times during the past week their child had consumed these foods outside preschool hours. The FFQ was intentionally restricted to not cover food consumption during the preschool hours since the parents would not have been able to reliably report it. For each food item, there were three answer options: ‘not at all’, ‘X times per day’ and ‘X times per week’. The parents were instructed to use the ‘not at all’ column or write the number in one of the other two answer columns that appeared more appropriate.

*A posteriori* data-derived dietary patterns were identified based on the FFQ data of 47 food groups using principal component analysis in our previous study (36). Principal component analysis combined the consumption of foods and beverages that correlated with each other (37). Briefly, three dietary patterns were identified based on the eigenvalue of ≥1.5, scree plot inspection and the reasonability of the pattern contents. Rerun with the three patterns was performed with an orthogonal Varimax rotation. Standardised pattern scores were calculated for each child. The dietary patterns were labelled ‘sweets-and-treats’, ‘health-conscious’ and ‘vegetables-and-processed meats’ and explained 16.7% of the variance in total (Table 1). The pattern score distributions were divided into groups of thirds that were labelled ‘low’, ‘moderate’ and ‘high’ adherence groups and used as predictors in this study.

**Table 1:** Description of the three dietary patterns identified in Finnish preschoolers participating in the DAGIS survey (2015–2016)

Sweets-and-treats pattern	Health-conscious pattern	Vegetables-and-processed meats pattern
Sweet biscuits and cereal bars (0.54)	Plain nuts, almonds and seeds (0.59)	Fresh vegetables (0.54)
Chocolate (0.53)	Natural yoghurt and quark (0.52)	Cold cuts (0.51)
Ice cream (0.50)	Berries (0.47)	Fresh fruit (0.40)
Sweets (0.47)	Egg (0.44)	Flavoured yoghurt and quark (0.40)
Soft drinks (0.47)	Wholegrain porridge, nonsweetened breakfast cereals and muesli (0.43)	Wholemeal bread (0.39)
Sugar-sweetened juice drinks (0.42)	Dried fruits and berries (0.41)	High-fat cheese (0.36)
Sweet pastries (0.38)	Wholegrain rice and pasta (0.40)	Fruit juice (0.36)
Crisps and popcorn (0.37)	Peas, beans, lentils and soya (0.33)	Sausages, frankfurters and luncheon meats (0.33)
Sugar-sweetened breakfast cereals and muesli (0.37)	Cooked and canned vegetables (0.32)	Berries (0.30)
Flavoured nuts, almonds and seeds (0.35)	Commercial baby foods and smoothies (0.31)	
Sausages, frankfurters and luncheon meats (0.31)		
Eigenvalue: 3.2	Eigenvalue: 2.8	Eigenvalue: 1.8
Percentage of total variance explained: 6.8%	Percentage of total variance explained: 6.1%	Percentage of total variance explained: 3.8%

Only food items loaded with an absolute value of ≥0.30 are shown. Absolute values are indicated in parentheses. For more detailed descriptions, see (36).

The FFQ has shown acceptable reproducibility (38) and validity against 3-day food records (39); it produced dietary patterns consistent with those derived from the food record data, and 72–73% of the children were classified into the same or adjacent quarters of the dietary pattern scores when the two methods were compared (39).

The FFQ also collected information on supplement use. The parents were instructed to specify which supplements, if any, their children had consumed during the past month. In the present study, we considered the supplemental intakes of vitamins A, C and D in addition to zinc, iron and probiotics. For each dietary supplement as well as for probiotic supplements, the children were divided into users (i.e., received more than 0 g/month of a particular nutrient or more than zero colony-forming units/month of any probiotics) and nonusers (i.e., received 0 g/month of a particular nutrient or zero colony-forming units/month of any probiotics).

### Outcome assessment

We had three outcomes: the number of common colds (hereafter colds), gastroenteritis episodes and antibiotic courses that the children had experienced during the past year. The outcomes were retrospectively reported by the parents completing the survey questionnaire (see Supplementary Table 1 for more detailed information). Neither definitions for the outcomes nor other detailed reporting guidelines were provided. In the descriptive analyses, each outcome was divided into two categories based on their frequency distributions and reasonability: for colds, into frequently affected (≥5 episodes) and less affected (<5 episodes) children; for gastroenteritis, into affected (≥1 episode) and unaffected (0 episodes) children; and for antibiotic courses, into consumers (≥1 course) and nonconsumers (0 courses). In multivariable modelling, colds and antibiotic courses were kept in count form due to their wide frequency distributions. Gastroenteritis remained classified into unaffected and affected children due to its narrow frequency distribution and the abundance of values 0 and 1 (47 and 42% of all observed values, respectively).

As the families attended the survey at different times of the year, we took into account the date on which the parents reported the outcomes, hereafter termed as the research season. We divided the research season into three categories: September–October, November–December and January–April.

### Covariates

Information about the following background factors was collected via the survey questionnaire completed by the parents: children’s age, sex and frequency of preschool attendance (days/week), as well as the number of children living in the same household, family’s highest education and family’s relative net incomes (euros/month). Age and family’s relative net incomes were used as continuous variables. The number of children living in the same household was divided into the following three categories in the descriptive analyses: (1) 0 children, (2) 1 child and (3) ≥2 children living in the same household. In multivariable modelling, this variable was used in countable form. Frequency of preschool attendance was divided into the following two categories in the descriptive analyses: (1) <5 days/week and (2) 5 days/week. In multivariable modelling, this was used as a countable variable. The parents reported their highest educational level among the following options: (1) comprehensive school, (2) vocational school, (3) high school, (4) bachelor’s degree or college, (5) master’s degree or (6) licentiate/doctor. These were further classified into the following three categories, the highest of which in the family was used in the analyses: (1) secondary school or lower, (2) bachelor’s degree or equivalent and (3) master’s degree or higher.

In the preschools, trained researchers measured children’s weight and height. Weight was measured using CAS portable bench scales (CAS PB-100/200) such that the children were without shoes and heavy clothing. Height was measured using stadiometers (SECA 217). Body mass index (BMI) was calculated as bodyweight (kg)/height^2^ (m). We used BMI as a continuous variable in the analyses. We also considered BMI as a categorical variable using extended international BMI cut-offs for weight status (underweight, normal weight, or overweight or obese) (40).

### Statistical methods

We used independent samples t-tests to compare continuous covariates and chi-square tests for independence to compare categorical covariates across the groups of binary outcomes (see the section ‘Outcome assessment’). Chi-square tests were used to compare the total number of infectious outcomes across the thirds of the dietary pattern scores. Logistic regression models were applied to assess the associations between thirds of the dietary pattern scores and the risk of experiencing gastroenteritis, with results shown as unadjusted and adjusted odds ratios (ORs) and their 95% confidence intervals (CIs). Negative binomial regression models were applied to assess the associations between thirds of the dietary pattern scores and the prevalence of colds and antibiotic courses, with results shown as unadjusted and adjusted prevalence ratios (PRs) and their 95% CIs. We chose negative binomial models because the preplanned Poisson models demonstrated overdispersion, a higher range of residuals, and higher values of Akaike’s and Bayesian information criteria. We also tested zero-inflated regression models for antibiotics due to a plethora of zeros in the distribution, but the negative binomial model tended to have a lower range of residuals and lower values of Akaike’s and Bayesian information criteria. The unadjusted models simultaneously included the thirds of all three dietary patterns of which low adherence groups were set as the reference. The adjusted models additionally included the following covariates: age, sex, frequency of preschool attendance, number of children living in the same household, family’s highest education and use of probiotic supplements. Details of these covariates are summarised in Supplementary Table 1. We tested but did not include the following covariates in the final models since their effects on the estimates were not substantial in magnitude (Supplementary Tables 2–4) and included plenty of missing values: the research season, dietary supplement use (nutrients), BMI, weight status and family’s relative net income. We performed sensitivity analyses using continuous natural log-transformed dietary pattern scores as predictors since the dietary pattern score distributions were not normally distributed (data not shown). We also fitted separate models for the thirds of each dietary pattern and obtained comparable results to the joint models (data not shown). All statistical analyses were performed at the 5% level of significance using RStudio version 2022.07.1 and R version 4.2.1. The functions *glm* and *glm.nb* from the package *MASS* were used for the logistic and negative binomial regression analyses, respectively.

## Results

### Participant characteristics

The current study included 721 children (83% of the final survey sample) with complete data on food consumption and gastroenteritis episodes. Compared with the excluded children who did not report the complete data (*n* = 143), the current study sample comprised a higher proportion of children who came from higher educated families and did not attend preschool 5 days a week (Supplementary Table 5). No other differences between excluded and included children were observed in terms of the examined variables.

Median number of gastroenteritis episodes was one (range: 0–5, interquartile range [IQR]: 0–1), of colds two (range: 0–15, IQR: 2–4) and of antibiotic courses zero (range: 0–11, IQR: 0–1). Nearly one-sixth of the children were affected by ≥5 colds during the past year (Table 2). Roughly half of the children were affected by ≥1 gastroenteritis episode, and nearly half had consumed ≥1 antibiotic course. The children affected by ≥5 colds and consuming ≥1 antibiotic course were younger on average than their less affected and nonconsuming peers. The children affected by ≥1 gastroenteritis episode and consuming ≥1 antibiotic course more likely attended preschool 5 days/week than their unaffected and nonconsuming peers. The children affected by ≥5 colds more likely used probiotics and came from the high-educated families than their less affected peers, but the latter difference did not reach statistical significance.

**Table 2:** Background information by the prevalence of common infections and antibiotic use among Finnish preschoolers (n = 706–721) participating in the DAGIS survey (2015–2016)

Background factors	All	Episodes of gastroenteritis during the past year			Episodes of common colds during the past year			Courses of antibiotics during the past year		
		0	≥1	*P*	0–4	≥5	*P*	0	≥1	*P*
*n* (%)	721 (100)	342 (47)	379 (53)		602 (83)	116 (16)		394 (55)	325 (45)	
Boys, *n* (%)	368 (51)	178 (52)	190 (50)	0.608	302 (50)	64 (55)	0.323	193 (49)	173 (53)	0.257
Age, years										
Mean ± SD	4.7 ± 0.89	4.7 ± 0.93	4.8 ± 0.86	0.239	**4.8 ± 0.90**	**4.4 ± 0.82**	**<0.001**	**4.8 ± 0.90**	**4.6 ± 0.88**	**0.012**
Preschool attendance, *n* (%)										
5 days/week	454 (63)	**198 (58)**	**256 (68)**	**0.008**	379 (63)	73 (63)	0.979	**235 (60)**	**218 (67)**	**0.044**
Missing	1 (0.14)	1 (0.29)	0 (0)		1 (0.16)	0 (0)		1 (0.25)	0 (0)	
Highest educational level in the family, *n* (%)				0.908			0.058			0.462
Secondary school or lower	151 (21)	71 (21)	80 (21)		126 (21)	24 (21)		80 (20)	70 (22)	
Bachelor’s degree or equivalent	304 (42)	147 (43)	157 (41)		264 (44)	39 (34)		175 (44)	129 (40)	
Master’s degree or higher	262 (36)	122 (36)	140 (37)		208 (35)	53 (46)		138 (35)	124 (38)	
Missing	4 (0.55)	2 (0.58)	2 (0.53)		4 (0.66)	0 (0)		1 (0.25)	2 (0.62)	
Number of children living in the same household, *n* (%)				0.179			0.256			0.069
0	95 (13)	52 (15)	43 (11)		77 (13)	18 (16)		45 (11)	50 (15)	
1	400 (55)	179 (52)	221 (58)		329 (55)	69 (60)		213 (54)	186 (57)	
≥2	226 (31)	111 (32)	115 (30)		196 (33)	29 (25)		136 (35)	89 (27)	
Used probiotic supplements, *n* (%)										
Yes	80 (11)	40 (12)	40 (11)	0.632	**57 (9.5)**	**22 (19)**	**0.003**	38 (9.6)	41 (13)	0.197
Missing	7 (0.97)	3 (0.88)	4 (1.1)		6 (1.0)	1 (0.86)		3 (0.76)	4 (1.2)	

Age was compared between the outcome categories using independent samples t-test. Categorical covariates were compared between the outcome categories using chi-square independence test. Missing observations are shown if they were present. Significant differences (*P* < 0.05) are indicated in boldface.

SD, Standard deviation.

Characteristics of the children also differed by adherence to the dietary patterns (Supplementary Table 6). For example, the children with low and moderate adherence to the sweets-and-treats pattern more likely came from high-educated families than their peers with high adherence (40, 40 and 29%, respectively; *P* = 0.038). Moreover, the children with high and moderate adherence to the health-conscious pattern more likely came from high-educated families than their peers with low adherence (44, 39 and 27%, respectively; *P* = 0.001).

### Dietary patterns in relation to common infections and antibiotic courses

Total number of colds and antibiotic courses differed significantly within the sweets-and-treats pattern, being highest in low adherence (Table 3), and within the health-conscious pattern, being highest in high adherence.

**Table 3:** Total number of parent-reported infection episodes and antibiotic courses by adherence to dietary patterns among Finnish preschoolers participating in the DAGIS survey (2015–2016)

Adherence to dietary patterns	*n*	Gastroenteritis, total number of episodes during the past year	Common colds, total number of episodes during the past year	Antibiotics, total number of courses during the past year
Sweets-and-treats				
Low	240	166	**764**	**216**
Moderate	241	150	**679**	**168**
High	240	152	**661**	**184**
*P*		0.614	**0.013**	**0.043**
Health-conscious				
Low	240	167	**664**	**172**
Moderate	241	151	**662**	**178**
High	240	150	**778**	**218**
*P*		0.558	**0.002**	**0.037**
Vegetables-and-processed meats				
Low	240	158	702	192
Moderate	241	136	701	177
High	240	174	701	199
*P*		0.097	0.999	0.513
In total	721	468	2,104	568

Total numbers of infection episodes and antibiotic courses across adherence groups were compared using the chi-square test assuming the expected proportion in each adherence group is 1/3. Significant differences (*P* < 0.05) are indicated in boldface.

In the unadjusted regression models, the risk of experiencing ≥1 gastroenteritis episode was lower in moderate adherence to the sweets-and-treats pattern than in low adherence (Table 4). Prevalence of colds was lower in high adherence to the sweets-and-treats pattern than in low adherence and higher in high adherence to the health-conscious pattern than in low adherence.

**Table 4:** Adherence to dietary patterns and their cross-sectional associations with common infections and antibiotic courses among Finnish preschoolers (n = 706–721) participating in the DAGIS survey (2015–2016)

Adherence to dietary patterns	Affected by gastroenteritis (≥1 episode) during the past year				Prevalence of common colds during the past year				Prevalence of antibiotic courses during the past year			
	OR (95% CI), unadjusted^a^	*P*	OR (95% CI), adjusted^b^	*P*	PR (95% CI), unadjusted^a^	*P*	PR (95% CI), adjusted^b^	*P*	PR (95% CI), unadjusted^a^	*P*	PR (95% CI), adjusted^b^	*P*
Sweets-and-treats												
Low	Ref.		Ref.		Ref.		Ref.		Ref.		Ref.	
Moderate	**0.68 (0.47**–**0.98)**	**0.038**	**0.63 (0.44**–**0.92)**	**0.016**	0.90 (0.80–1.00)	0.060	**0.89 (0.80**–**1.00)**	**0.047**	0.80 (0.61–1.05)	0.100	**0.77 (0.59**–**1.00)**	**0.047**
High	0.78 (0.54–1.13)	0.186	0.75 (0.51–1.09)	0.130	**0.86 (0.77**–**0.97)**	**0.013**	**0.88 (0.79**–**0.99)**	**0.032**	0.86 (0.66–1.12)	0.260	0.89 (0.69–1.15)	0.375
Health-conscious												
Low	Ref.		Ref.		Ref.		Ref.		Ref.		Ref.	
Moderate	0.85 (0.59–1.22)	0.379	0.85 (0.59–1.24)	0.399	0.99 (0.88–1.11)	0.807	0.98 (0.87–1.10)	0.737	1.02 (0.78–1.34)	0.880	1.04 (0.79–1.35)	0.798
High	0.74 (0.51–1.06)	0.099	0.78 (0.53–1.14)	0.199	**1.16 (1.03**–**1.30)**	**0.012**	**1.13 (1.01**–**1.27)**	**0.038**	1.22 (0.93–1.59)	0.140	1.18 (0.90–1.54)	0.232
Vegetables-and-processed meats												
Low	Ref.		Ref.		Ref.		Ref.		Ref.		Ref.	
Moderate	0.79 (0.55–1.14)	0.213	0.75 (0.52–1.09)	0.135	0.99 (0.89–1.12)	0.979	1.01 (0.90–1.13)	0.874	0.94 (0.72–1.22)	0.620	0.93 (0.71–1.21)	0.576
High	1.23 (0.86–1.78)	0.257	1.26 (0.86–1.83)	0.235	1.00 (0.89–1.12)	0.974	1.02 (0.91–1.15)	0.710	1.03 (0.80–1.34)	0.800	1.09 (0.84–1.41)	0.528
* n*	721		709		718		706		719		708	

Logistic regression analysis was used for gastroenteritis. Negative binomial regression analysis was used for common colds and antibiotic courses.

Significant associations (*P* < 0.05) are indicated in boldface.

a The thirds of all three dietary patterns were entered to the model simultaneously.

b Adjusted for age (years), sex, number of children living in the same household, highest educational level in the family (secondary school or lower, bachelor’s degree or equivalent, or master’s degree or higher), frequency of preschool attendance (days/week) and use of probiotic supplements (yes or no).

CI, Confidence interval; OR, Odds ratio; PR, Prevalence ratio; Ref., Reference group.

After adjusting for age, sex, number of children living in the same household, frequency of preschool attendance, highest education in the family and probiotic use, the risk of experiencing ≥1 gastroenteritis episode remained lower in moderate adherence to the sweets-and-treats pattern than in low adherence (Table 4). Prevalence of colds was lower in both moderate and high adherence to the sweets-and-treats pattern than in low adherence and was higher in high adherence to the health-conscious pattern than in low adherence. Moreover, prevalence of antibiotic courses was lower in moderate adherence to the sweets-and-treats pattern than in low adherence. Prevalence of infectious outcomes did not differ significantly within the categories of adherence to the vegetables-and-processed meats pattern.

### Sensitivity analyses

Sensitivity analyses using continuous log-transformed dietary pattern scores as predictors produced rather similar results. The sweets-and-treats pattern scores were inversely associated with the prevalence of colds (unadjusted PR: 0.80, 95% CI: 0.70–0.93, *P* = 0.003; adjusted PR: 0.83, 95% CI: 0.72–0.96, *P* = 0.014). Health-conscious pattern scores tended to be positively associated with the prevalence of colds (unadjusted PR: 1.19, 95% CI: 1.03–1.39, *P* = 0.023; adjusted PR: 1.15, 95% CI: 0.99–1.35, *P* = 0.078). No other significant associations were observed.

## Discussion

The results of this cross-sectional study among Finnish preschoolers contradicted the hypothesised outcome. The sweets-and-treats pattern was inversely associated with the prevalence of colds, while the health-conscious pattern showed a positive association. Also, the sweets-and-treats pattern tended to be inversely associated with the risk of experiencing ≥1 gastroenteritis episode and the prevalence of antibiotic courses. No significant associations between the vegetables-and-processed meats pattern and infectious outcomes were found.

Our results were not plausible from a biological viewpoint, nor were they coherent with the concept of overall health promotion. Frequent consumption of foods included in the health-conscious pattern can increase the intakes of polyunsaturated fatty acids, fibre, zinc, iron and vitamins A, D and C, all of which are essential in immune defence (10–15). Presumably, frequent consumption of sweets and snacks included in the sweets-and-treats pattern does not increase the nutrient density or fibre content of the diet but may replace healthier options. Also, sweets and snacks tend to be high in sugar and fat, particularly saturated fat. Excess sugar and fat *per se* may impair the mucosal epithelial barrier function in the gut (18, 19). Saturated fatty acids may additionally stimulate innate immune cells to induce unnecessary inflammation (19, 20).

Our results also contradicted previous research on preschoolers’ overall diet and infections. An earlier cross-sectional study among Finnish preschoolers demonstrated an inverse association between a dietary pattern emphasising fruit and berry consumption and the prevalence of acute otitis media (33). The study also found a positive association between a dietary pattern emphasising biscuit and sweet pastry consumption and the risk of pneumococcal carriage (33). However, the previous study extracted altogether nine dietary patterns, although the content of all of them did not seem meaningful; the proportions of variances or the absolute values of food loadings were also not presented (33). It has been recommended to retain fewer patterns to obtain meaningful pattern contents (41). Compared with the previous study’s nine dietary patterns, our study detected three dietary patterns that demonstrated reproducible contents (36). Nevertheless, the previous study found plausible associations, probably due to the fact the parents reported physician-diagnosed episodes of acute otitis media, and biological samples were taken (33). By contrast, our parents retrospectively reported the outcomes without provision of specific reporting guidelines or physician confirmation. These limitations may have affected our results.

Our main finding was that healthier dietary habits were associated with higher prevalence of colds and unhealthier diets with lower prevalence. Gastroenteritis episodes and antibiotic use tended to show similar associations. Previous studies have reported that parents’ healthy eating attitudes, nutritional knowledge and concerns about disease prevention can be related to their children’s healthier diets (42–44). Moreover, given that both recalling colds and gastroenteritis episodes and seeking antibiotics required parental effort, we presume our unexpected results might derive from parental behaviour, that is, the most health-conscious parents might recall symptoms suggesting colds and gastroenteritis and seek care for illnesses more vigorously than their less health-conscious peers. We noted that children with healthier dietary habits mostly came from high-educated families. Likewise, the children frequently sustaining colds tended to mostly come from high-educated families. In another cross-sectional study of Finnish preschoolers, both maternal and paternal academic degrees were linked to a higher risk of parent-reported recurrent respiratory illness relative to nonacademic degrees in univariable models, and the same was true for mothers in the multivariable stepwise model (45). The authors suggested that the difference was due to recall bias (45). Another Finnish study reported that higher educated parents had more positive attitudes towards using medicines for their children under 12 years of age than lower educated parents (46). Here, however, adjustment for the family’s highest education or the other covariates did not affect the estimates. On the other hand, 80% of the children in our study came from academically educated families. The corresponding proportions were 25% for mothers and 31% for fathers in the previous study finding positive relationships between parental education and respiratory illness (45). In our study, there may not have been enough variation in educational levels to detect their possible confounding role. Also, more research is warranted to determine which indicators of socioeconomic status are most consistently related to infectious outcomes. Parental working status and household’s income level have also been linked to Finnish parents’ attitudes towards using medication for their children (46). Nevertheless, to the best of our knowledge, previous research on the topic is sparse, and similar associations to ours have not been reported. Our interpretation of the results thus remains speculative. We also acknowledge the possibility that the results were due to chance since the magnitude of the associations observed was generally modest, mainly of borderline statistical significance.

We found no associations between the vegetables-and-processed meats pattern and the infectious outcomes. This dietary pattern included food items rich in micronutrients and fibre as well as those typically high in fat, particularly saturated fat. Somewhat similarly, among Dutch 1–4-year-old children suffering from recurrent upper respiratory infections, a 6-month dietary intervention aimed at frequent consumption of green vegetables, beef, whole milk and butter did not reduce the number of upper respiratory infections compared with habitual food consumption (47). In our study, habitual consumption of both beneficial and potentially harmful food items might neutralise the contribution of the vegetables-and-processed meats pattern to immunity. In fact, as a diet typically consists of both healthier and less healthy choices, one should be aware of the possibility that dietary habits may not ultimately play a significant role in preschoolers’ susceptibility to infections if well-nourished *per se*. We previously reported that our study population comprised mainly normal weight children (81% of 3–4-year-olds and 79% of 5–6-year-olds) (48), and here, test adjustments for BMI and weight status did not significantly modify our results. However, underweight children of Western countries may be at risk of infections, while the risk is less clear in overweight and obese children (49). Weight status may be linked to immune dysfunction through increased inflammation and impaired regulation of immune responses (50). Future studies addressing the role of weight status may aid in elucidating the relation between diet and infections in preschoolers of Western countries.

A strength of our study includes the relatively large sample size. Moreover, we focused on children’s diet as a whole, instead of single nutrients or food items. Individual dietary components might not have had the power to similarly reveal the observed associations (30, 31). Furthermore, the dietary patterns were based on the FFQ showing acceptable validity (39) and reproducibility (38). We used a 1-week reference period for our FFQ to estimate children’s usual consumption frequency. Since FFQs rely on generic rather than episodic memory (51, 52) and recalling food consumption even for the previous day can be demanding and introduce recall bias (53, 54), we considered parents’ retrospective reports for the previous week to be applicable to describe children’s usual food consumption outside preschool hours. In addition, we looked at the associations at the dietary pattern level. It has been shown that dietary patterns are relatively stable throughout childhood (55). However, since the FFQ did not cover food consumption during preschool hours, the dietary patterns did not reflect the children’s overall diet. This was intentional since the parents could not have reliably assessed their children’s food consumption during preschool hours. The previous study seemed to measure preschoolers’ overall diet using an FFQ; however, the authors did not discuss the reliability of assessing food consumption during preschool hours (33). We acknowledge that reliable measures of food consumption during preschool hours and their inclusion in the analysis can give a more accurate picture of the importance of the whole diet for infections. Nevertheless, in Finland, the municipality offers three free meals during the preschool day to all children. Daily meals are offered regularly, and each meal should consist of certain food groups (56). It is also recommended that the menus are rotated at regular intervals (56). Therefore, the greatest variation in the dietary habits of Finnish preschoolers presumably derives from foods consumed outside preschool hours. In addition, our results would have been based on a biased number of infectious episodes and antibiotic courses regardless of whether the food consumption during preschool hours had been included in the dietary pattern analysis.

The generalisability of our results is somewhat limited since 80% of the children came from families with at least a bachelor’s degree or equivalent. In Finland overall, 39% of 30–34-year-olds, 44% of 35–39-year-olds and 47% of 40–44-year-olds have at least a bachelor’s degree or equivalent (57). Furthermore, due to our cross-sectional design, conclusions about causality cannot be drawn, and the possibility of reverse causality should be considered; parents whose children were frequently affected by infections during the preceding year might later improve the quality of their child’s diet. Also, a 1-year retrospective reporting period for the infectious outcomes might cause recall bias, and the lack of adequate reporting guidelines has likely allowed parents to interpret symptoms differently, both exposing our results to misreporting. More reliable information on the use of antibiotics would have been obtained from the national prescription register, but unfortunately, the data were not available for the children in our study. Moreover, possible contribution of other factors beyond our control must be acknowledged; the epidemiology of infections can be multifactorial (1, 2, 45, 58), and dietary patterns can have strong interactions with many lifestyle traits (37). Nevertheless, we could not record the infectious outcomes and related risk factors as comprehensively as the current topic would have required since the DAGIS study was primarily designed to evaluate factors associated with energy balance-related behaviour in preschoolers (34, 35). Other limitations include subjective decision-making being present when identifying dietary patterns and the dietary patterns combined explaining a relatively low proportion of the children’s food consumption.

## Conclusions

The results of this study in Finnish preschoolers were unexpected. A healthier dietary pattern was associated with higher prevalence of colds, while an unhealthier dietary pattern showed lower associations. A similar tendency was observed for antibiotic use and gastroenteritis. Parents who were more health-conscious of their children’s diet might also be more aware of their children’s illness than less health-conscious parents.

Nevertheless, previous research on the topic appeared sparse. Further investigation is warranted to resolve whether dietary habits contribute to susceptibility to infections in preschoolers of Western countries. To obtain valid infectious outcomes, a prospective design should be implemented and objective measures or adequate reporting guidelines should be considered.

## Supplementary Material

Associations of dietary patterns with common infections and antibiotic use among Finnish preschoolers
