# Supplementary material for: Associations of dietary patterns with common infections and antibiotic use among Finnish preschoolers
Source: Food Nutr Res. 2023 Jun 14;67:10.29219/fnr.v67.8997. doi: 10.29219/fnr.v67.8997 (PMC10284098; doi:10.29219/fnr.v67.8997)
Supplement: Associations of dietary patterns with common infections and antibiotic use among Finnish preschoolers [file FNR-67-8997-s001.docx]

**Supplementary material**

**Article title** Associations of Dietary Patterns with Common Infections and Antibiotic Use among Finnish Preschoolers

**Journal name** Food & Nutrition Research

**Contents**

**Table S1** Details of the outcomes and covariates included in the final regression models of the associations of dietary patterns with common infections and antibiotic use among Finnish preschoolers participating in the DAGIS survey (2015–2016).

**Table S2** Final logistic regression model on the associations of dietary patterns with the risk of gastroenteritis and test adjustments for additional covariates.

**Table S3** Final negative binomial regression model on the associations of dietary patterns with the prevalence of common colds and test adjustments for additional covariates.

**Table S4** Final negative binomial regression model on the associations of dietary patterns with the prevalence of antibiotic courses and test adjustments for additional covariates.

**Table S5** Differences between the included and excluded children in the study of the associations between dietary patterns, common infections, and antibiotic use among Finnish preschoolers. DAGIS survey (2015–2016).

(continued)

**Table S6** Background information by adherence to the dietary patterns among Finnish preschoolers in the DAGIS survey (2015–2016).

**Table S1** Details of the outcomes and covariates included in the final regression models of the associations of dietary patterns with common infections and antibiotic use among Finnish preschoolers participating in the DAGIS survey (2015–2016).

(continued)

| Role of variable | Variable | Survey question | Answer options | Type of variable in the descriptive analyses; categories if applicable | Type of variable in multivariable modelling; categories if applicable |
| --- | --- | --- | --- | --- | --- |
| Outcome | Common colds | How many episodes of common colds has your child experienced during the past year? | Numeric answer | Classified; a. slightly affected (0–4 episodes) b. frequently affected (≥ 5 episodes) | Countable |
|  | Gastroenteritis | How many episodes of gastroenteritis has your child experienced during the past year? | Numeric answer | Classified; a. unaffected (0 episodes) b. affected (≥ 1 episode) | Classified; a. unaffected (0 episodes) b. affected (≥ 1 episode) |
|  | Antibiotic courses | How many courses of antibiotics has your child consumed during the past year? | Numeric answer | Classified; a. non-consumers (0 courses) b. consumers (≥ 1 course) | Countable |
| Covariate | Age | Age of the participating children as years and months | Numeric answers | Continuous | Continuous |
|  | Sex | - | - | Classified; a. boys b. girls | Classified; a. boys b. girls |
|  | Preschool attendance | How many days a week does your child attend preschool? | Numeric answer | Classified; a. children attending < 5 days a week b. children attending 5 days a week | Countable |
|  | Number of children living in the same household | How many persons live in your household at the moment? | Numeric answers requested for i. adults  ii. children ≥ 18 years iii. children aged 7–17 years iv. children aged 3–6 years v. children aged 0–2 years.  Only questions iii-v included and their answers were combined in the current analyses. | Classified; a. 0 b. 1 c. ≥ 2 | Countable |
|  | Highest educational level in the family | What is the highest degree of education you/your partner have/has achieved? | a. comprehensive school b. vocational school c. high school d. bachelor’s degree or equivalent e. master’s degree f. licentiate/doctor g. other | Classified; a. secondary school or lower b. bachelor’s degree or equivalent c. master’s degree or higher | Classified; a. secondary school or lower b. bachelor’s degree or equivalent c. master’s degree or higher |
|  | Probiotic use | During the past month, has your child used supplements? | Answers requested for i. the name of the supplement(s)  ii. the dose(s) of the supplement(s)  iii. the intake frequency(/-ies) of the supplement(s) | Classified; a. children who did not use probiotics b. children who used ≥ 1 probiotic supplement despite the probiotic strain. | Classified; a. children who did not use probiotics b. children who used ≥ 1 probiotic supplement despite the probiotic strain. |

**Table S1** (continued)

**Table S2** Final logistic regression model on the associations of dietary patterns with the risk of gastroenteritis and test adjustments for additional covariates. The additional covariates were not simultaneously included in the model. DAGIS survey among Finnish preschoolers (2015–2016).

|  | Affected by gastroenteritis (1 episode or more) during the past year,  OR (95% CI) | | | | | | | | | |
| --- | --- | --- | --- | --- | --- | --- | --- | --- | --- | --- |
| Adherence  to dietary patterns | final adjusted model ^a^ | further adjusted for research season ^b^ | further adjusted for BMI (kg/m^2^) | further adjusted for weight status ^c^ | further adjusted for family’s relative net incomes (euros/month) | further adjusted for supplemental vitamin A ^d^ | further adjusted for supplemental vitamin C ^d^ | further  adjusted for  supplemental vitamin D ^d^ | further adjusted for supplemental zinc ^d^ | further adjusted for supplemental iron ^d^ |
| *Sweets-and-treats* | |  |  |  |  |  |  |  |  |  |
| Low | Ref. | Ref. | Ref. | Ref. | Ref. | Ref. | Ref. | Ref. | Ref. | Ref. |
| Moderate | **0.63**  **(0.44**–**0.92)** | **0.63**  **(0.44**–**0.92)** | **0.58**  **(0.39**–**0.86)** | **0.61**  **(0.41**–**0.89)** | **0.66**  **(0.44**–**0.98)** | **0.64**  **(0.44**–**0.94)** | **0.63**  **(0.43**–**0.92)** | **0.62**  **(0.43**–**0.91)** | **0.63**  **(0.43**–**0.92)** | **0.63**  **(0.43**–**0.92)** |
| High | 0.75  (0.51–1.09) | 0.74  (0.51–1.08) | 0.70  (0.48–1.04) | 0.73  (0.49–1.07) | 0.80  (0.53–1.20) | 0.74  (0.50–1.08) | 0.74  (0.50–1.08) | 0.71  (0.49–1.05) | 0.73  (0.50–1.07) | 0.72  (0.49–1.06) |
| *Health-conscious* | |  |  |  |  |  |  |  |  |  |
| Low | Ref. | Ref. | Ref. | Ref. | Ref. | Ref. | Ref. | Ref. | Ref. | Ref. |
| Moderate | 0.85  (0.59–1.24) | 0.86  (0.59–1.24) | 0.75  (0.51–1.11) | 0.76  (0.52–1.12) | 0.79  (0.53–1.17) | 0.86  (0.58–1.25) | 0.86  (0.59–1.25) | 0.85  (0.58–1.25) | 0.85  (0.58–1.23) | 0.84  (0.58–1.23) |
| High | 0.78  (0.53–1.14) | 0.79  (0.54–1.17) | 0.71  (0.48–1.06) | 0.72  (0.48–1.07) | 0.78  (0.51–1.18) | 0.77  (0.52–1.14) | 0.78  (0.53–1.15) | 0.78  (0.53–1.15) | 0.77  (0.52–1.14) | 0.77  (0.52–1.13) |
| *Vegetables-and-processed meats* | | |  |  |  |  |  |  |  |  |
| Low | Ref. | Ref. | Ref. | Ref. | Ref. | Ref. | Ref. | Ref. | Ref. | Ref. |
| Moderate | 0.75  (0.52–1.09) | 0.74  (0.51–1.08) | 0.77  (0.52–1.13) | 0.78  (0.53–1.15) | 0.78  (0.53–1.17) | 0.74  (0.51–1.09) | 0.76  (0.52–1.10) | 0.76  (0.52–1.11) | 0.76  (0.52–1.11) | 0.76  (0.52–1.11) |
| High | 1.26  (0.86–1.83) | 1.24  (0.85–1.81) | 1.30  (0.88–1.92) | 1.30  (0.88–1.92) | 1.42  (0.94–2.16) | 1.27  (0.87–1.86) | 1.28  (0.87–1.87) | 1.28  (0.88–1.88) | 1.29  (0.88–1.89) | 1.28  (0.88–1.88) |
| *n* | 709 | 709 | 668 | 668 | 614 | 682 | 682 | 682 | 682 | 682 |
| total % of correctly classified | 60 | 58 | 58 | 59 | 59 | 58 | 58 | 58 | 59 | 58 |

Significant associations (*P*<0.05) are indicated in boldface.

^a^ Adjusted for age (years), sex, number of children living in the same household, highest educational level in the family (secondary school or lower, bachelor’s degree or equivalent, or master’s degree or higher), frequency of preschool attendance (days/week), and use of probiotic supplements (yes or no). The thirds of all three dietary patterns were entered to the model simultaneously.

^b^ Sept–Oct, Nov–Dec, or Jan–Apr.

^c^ Underweight, normal weight, or overweight or obese [Cole TJ, Lobstein T. Extended international (IOTF) body mass index cut-offs for thinness, overweight and obesity. Pediatr Obes 2012; 7: 284–94. <https://doi.org/10.1111/j.2047-6310.2012.00064.x>]

^d^ Used or did not use.

CI, Confidence interval; OR, Odds ratio; Ref., Reference group.

**Table S3** Final negative binomial regression model on the associations of dietary patterns with the prevalence of common colds and test adjustments for additional covariates. The additional covariates were not simultaneously included in the model. DAGIS survey among Finnish preschoolers (2015–2016).

|  | Prevalence of common colds during the past year,  PR (95% CI) | | | | | | | | | |
| --- | --- | --- | --- | --- | --- | --- | --- | --- | --- | --- |
| Adherence to dietary patterns | final adjusted model ^a^ | further adjusted for research season ^b^ | further adjusted for BMI (kg/m^2^) | further adjusted for weight status ^c^ | further adjusted for family’s relative net incomes (euros/month) | further adjusted for supplemental vitamin A ^d^ | further adjusted for supplemental vitamin C ^d^ | further adjusted for supplemental vitamin D ^d^ | further adjusted for supplemental zinc ^d^ | further adjusted for supplemental iron ^d^ |
| *Sweets-and-treats* | |  |  |  |  |  |  |  |  |  |
| Low | Ref. | Ref. | Ref. | Ref. | Ref. | Ref. | Ref. | Ref. | Ref. | Ref. |
| Moderate | **0.89**  **(0.80–1.00)** | 0.89  (0.80–1.00) | **0.87**  **(0.77–0.98)** | **0.87**  **(0.77–0.98)** | **0.88**  **(0.78–0.99)** | 0.89  (0.80–1.00) | **0.89**  **(0.79–1.00)** | **0.89**  **(0.79–1.00)** | **0.89**  **(0.79–1.00)** | 0.89  (0.79–1.00) |
| High | **0.88**  **(0.79–0.99)** | **0.88**  **(0.79–0.99)** | 0.90  (0.80–1.01) | **0.88**  **(0.78–0.99)** | **0.87**  **(0.77–0.99)** | 0.90  (0.80–1.01) | 0.90  (0.80–1.01) | 0.89  (0.79–1.01) | 0.89  (0.79–1.01) | 0.90  (0.80–1.01) |
| *Health-conscious* | |  |  |  |  |  |  |  |  |  |
| Low | Ref. | Ref. | Ref. | Ref. | Ref. | Ref. | Ref. | Ref. | Ref. | Ref. |
| Moderate | 0.98  (0.87–1.10) | 0.98  (0.87–1.11) | 0.96  (0.85–1.08) | 0.96  (0.85–1.08) | 0.98  (0.86–1.10) | 0.98  (0.87–1.11) | 0.98  (0.87–1.10) | 0.98  (0.87–1.11) | 0.98  (0.87–1.10) | 0.98  (0.87–1.10) |
| High | **1.13**  **(1.01–1.27)** | **1.13**  **(1.01–1.27)** | 1.12  (0.99–1.26) | 1.11  (0.98–1.25) | 1.11  (0.98–1.26) | **1.14**  **(1.02–1.29)** | **1.14**  **(1.01–1.29)** | **1.14**  **(1.01–1.29)** | **1.14**  **(1.01–1.29)** | **1.14**  **(1.01–1.29)** |
| *Vegetables-and-processed meats* | | |  |  |  |  |  |  |  |  |
| Low | Ref. | Ref. | Ref. | Ref. | Ref. | Ref. | Ref. | Ref. | Ref. | Ref. |
| Moderate | 1.01  (0.90–1.13) | 1.01  (0.90–1.13) | 1.02  (0.90–1.15) | 1.01  (0.89–1.13) | 0.98  (0.87–1.11) | 0.99  (0.88–1.11) | 0.99  (0.88–1.12) | 0.99  (0.88–1.12) | 0.99  (0.88–1.12) | 0.99  (0.88–1.12) |
| High | 1.02  (0.91–1.15) | 1.02  (0.91–1.15) | 1.04  (0.93–1.18) | 1.04  (0.92–1.17) | 1.04  (0.92–1.18) | 1.01  (0.90–1.14) | 1.01  (0.90–1.14) | 1.01  (0.90–1.14) | 1.01  (0.90–1.14) | 1.02  (0.90–1.14) |
| *n* | 706 | 706 | 665 | 665 | 612 | 679 | 679 | 679 | 679 | 679 |

Significant associations (*P*<0.05) are indicated in boldface.

^a^ Adjusted for age (years), sex, number of children living in the same household, highest educational level in the family (secondary school or lower, bachelor’s degree or equivalent, or master’s degree or higher), frequency of preschool attendance (days/week), and use of probiotic supplements (yes or no). The thirds of all three dietary patterns were entered to the model simultaneously.

^b^ Sept–Oct, Nov–Dec, or Jan–Apr.

^c^ Underweight, normal weight, or overweight or obese [Cole TJ, Lobstein T. Extended international (IOTF) body mass index cut-offs for thinness, overweight and obesity. Pediatr Obes 2012; 7: 284–94. <https://doi.org/10.1111/j.2047-6310.2012.00064.x>.]

^d^ Used or did not use.

CI, Confidence interval; PR, Prevalence ratio; Ref., Reference group.

**Table S4** Final negative binomial regression model on the associations of dietary patterns with the prevalence of antibiotic courses and test adjustments for additional covariates.

The additional covariates were not simultaneously included in the model. DAGIS survey among Finnish preschoolers (2015–2016).

|  |  | Prevalence of antibiotic courses during the past year,  PR (95% CI) | | | | | | | | | |
| --- | --- | --- | --- | --- | --- | --- | --- | --- | --- | --- | --- |
| Adherence  to dietary patterns | final adjusted model ^a^ | | further adjusted for research season ^b^ | further adjusted for BMI (kg/m^2^) | further adjusted for weight status ^c^ | further adjusted for family’s relative net incomes (euros/month) | further adjusted for supplemental vitamin A ^d^ | further adjusted for supplemental vitamin C ^d^ | further adjusted for supplemental vitamin D ^d^ | further adjusted for supplemental zinc ^d^ | further adjusted for supplemental iron ^d^ |
| *Sweets-and-treats* | | |  |  |  |  |  |  |  |  |  |
| Low | Ref. | | Ref. | Ref. | Ref. | Ref. | Ref. | Ref. | Ref. | Ref. | Ref. |
| Moderate | **0.77**  **(0.59–1.00)** | | 0.78  (0.60–1.01) | 0.78  (0.59–1.02) | 0.78  (0.59–1.02) | **0.73**  **(0.55–0.96)** | **0.76**  **(0.58–0.99)** | **0.75**  **(0.57–0.98)** | **0.76**  **(0.58–1.00)** | **0.75**  **(0.57–0.98)** | **0.76**  **(0.58–0.99)** |
| High | 0.89  (0.69–1.15) | | 0.90  (0.69–1.16) | 0.91  (0.70–1.19) | 0.93  (0.71–1.21) | 0.93  (0.71–1.23) | 0.92  (0.71–1.20) | 0.89  (0.68–1.16) | 0.92  (0.71–1.20) | 0.89  (0.68–1.16) | 0.92  (0.71–1.20) |
| *Health-conscious* | | |  |  |  |  |  |  |  |  |  |
| Low | Ref. | | Ref. | Ref. | Ref. | Ref. | Ref. | Ref. | Ref. | Ref. | Ref. |
| Moderate | 1.04  (0.79–1.35) | | 1.05  (0.80–1.38) | 0.98  (0.75–1.30) | 0.98  (0.75–1.29) | 1.00  (0.76–1.33) | 1.06  (0.81–1.40) | 1.04  (0.79–1.36) | 1.04  (0.79–1.37) | 1.05  (0.80–1.38) | 1.06  (0.81–1.38) |
| High | 1.18  (0.90–1.54) | | 1.19  (0.90–1.55) | 1.17  (0.89–1.54) | 1.17  (0.89–1.55) | 1.13  (0.85–1.51) | 1.19  (0.90–1.56) | 1.17  (0.89–1.54) | 1.17  (0.89–1.54) | 1.18  (0.89–1.55) | 1.18  (0.90–1.56) |
| *Vegetables-and-processed meats* | | | |  |  |  |  |  |  |  |  |
| Low | Ref. | | Ref. | Ref. | Ref. | Ref. | Ref. | Ref. | Ref. | Ref. | Ref. |
| Moderate | 0.93  (0.71–1.21) | | 0.93  (0.71–1.21) | 0.92  (0.70–1.21) | 0.93  (0.71–1.22) | 0.80  (0.60–1.06) | 0.92  (0.71–1.21) | 0.94  (0.72–1.23) | 0.93  (0.71–1.22) | 0.93  (0.71–1.22) | 0.94  (0.72–1.23) |
| High | 1.09  (0.84–1.41) | | 1.08  (0.83–1.40) | 1.12  (0.86–1.46) | 1.13  (0.86–1.48) | 1.04  (0.79–1.37) | 1.09  (0.84–1.42) | 1.10  (0.85–1.44) | 1.10  (0.85–1.43) | 1.09  (0.84–1.42) | 1.12  (0.86–1.45) |
| *n* | 708 | | 708 | 667 | 667 | 613 | 681 | 681 | 681 | 681 | 681 |

Significant associations (*P*<0.05) are indicated in boldface.

^a^ Adjusted for age (years), sex, number of children living in the same household, highest educational level in the family (secondary school or lower, Bachelor’s degree or equivalent, or Master’s degree or higher), frequency of preschool attendance (days/week), and use of probiotic supplements (yes or no). The thirds of all three dietary patterns were entered to the model simultaneously.

^b^ Sept–Oct, Nov–Dec, or Jan–Apr.

^c^ Underweight, normal weight, or overweight or obese [Cole TJ, Lobstein T. Extended international (IOTF) body mass index cut-offs for thinness, overweight and obesity. Pediatr Obes 2012; 7: 284–94. <https://doi.org/10.1111/j.2047-6310.2012.00064.x>.]

^d^ Used or did not use.

CI, Confidence interval; PR, Prevalence ratio; Ref., Reference group.

**Table S5** Differences between the included and excluded children in the study of the associations between dietary patterns, common infections, and antibiotic use among Finnish preschoolers. DAGIS survey (2015–2016).

(continued)

| Role of variable | Variable | Whole survey sample | Included children | Excluded children | *P* |
| --- | --- | --- | --- | --- | --- |
|  | Sample size, *n* (%) | 864 (100) | 721 (83) | 143 (17) |  |
| Dietary pattern | Sweets-and-treats scores ^a^, mean (SD) | 0.00 (1.00) | -0.01 (1.00) | 0.24 (0.98) | 0.142 |
|  | Missing, *n* (%) | 106 (12) | 0 (0) | 106 (74) |  |
|  | Health-conscious scores ^a^, mean (SD) | 0.00 (1.00) | -0.01 (0.94) | 0.27 (1.81) | 0.353 |
|  | Missing, *n* (%) | 106 (12) | 0 (0) | 106 (74) |  |
|  | Vegetables-and-processed meats scores ^a^, mean (SD) | 0.00 (1.00) | -0.00 (0.99) | 0.04 (1.20) | 0.808 |
|  | Missing, *n* (%) | 106 (12) | 0 (0) | 106 (74) |  |
| Infectious outcomes | Gastroenteritis episodes during the past year: |  |  |  |  |
|  | 1 or more, *n* (%) | 418 (48) | 379 (53) | 39 (27) | 0.836 |
|  | Missing, *n* (%) | 67 (7.8) | 0 (0) | 67 (47) |  |
|  | Common cold episodes during the past year: |  |  |  |  |
|  | 5 or more, *n* (%) | 127 (15) | 116 (16) | 11 (7.7) | 0.670 |
|  | Missing, *n* (%) | 69 (8.0) | 3 (0.42) | 66 (46) |  |
|  | Antibiotic courses during the past year: |  |  |  |  |
|  | 1 or more, *n* (%) | 365 (42) | 325 (45) | 40 (28) | 0.259 |
|  | Missing, *n* (%) | 68 (7.9) | 2 (0.28) | 66 (46) |  |
| Covariates | Sex: |  |  |  |  |
|  | Boys, *n* (%) | 450 (52) | 368 (51) | 82 (57) | 0.144 |
|  | Missing, *n* (%) | 1 (0.12) | 0 (0) | 1 (0.70) |  |
|  | Age (years), mean (SD) | 4.7 (0.90) | 4.7 (0.89) | 4.8 (0.91) | 0.463 |
|  | Missing, *n* (%) | 0 (0) | 0 (0) | 0 (0) |  |
|  | Preschool attendance: |  |  |  |  |
|  | 5 days/week, *n* (%) | 515 (60) | **454 (63)** | **61 (43)** | **0.029** |
|  | Missing, *n* (%) | 63 (7.3) | 1 (0.14) | 62 (43) |  |
|  | Highest educational level in the family: |  |  |  | **0.002** |
|  | Secondary school or lower, *n* (%) | 200 (23) | **151 (21)** | **49 (34)** |  |
|  | Bachelor’s degree or equivalent, *n* (%) | 356 (41) | **304 (42)** | **52 (36)** |  |
|  | Master’s degree or higher, *n* (%) | 303 (35) | **262 (36)** | **41 (29)** |  |
|  | Missing, *n* (%) | 5 (0.58) | 4 (0.55) | 1 (0.70) |  |
|  | Number of children living in the same household: |  |  |  | 0.833 |
|  | 0, *n* (%) | 104 (12) | 95 (13) | 9 (6.3) |  |
|  | 1, *n* (%) | 447 (52) | 400 (55) | 47 (33) |  |
|  | 2 or more, *n* (%) | 253 (29) | 226 (31) | 27 (19) |  |
|  | Missing, *n* (%) | 60 (6.9) | 0 (0) | 60 (42) |  |
|  | Used probiotic supplements: |  |  |  | 0.731 |
|  | Yes, *n* (%) | 89 (10) | 80 (11) | 9 (6.3) |  |
|  | Missing, *n* (%) | 60 (6.9) | 7 (0.97) | 53 (37) |  |

Age and dietary patterns scores were compared between the included and excluded children using independent samples t-tests. Categorical covariates were compared between the included and excluded children using chi-square independence tests. Significant differences (*P*<0.05) are indicated in boldface.

**Table S5** (continued)

^a^ Used as a continuous variable since the included children would have been ranked differently if adherence to the thirds of the dietary pattern scores had been assessed among the whole survey sample.

SD, Standard deviation.

**Table S6** Background information by adherence to the dietary patterns among Finnish preschoolers in the DAGIS survey (2015–2016).

(continued)

|  |  | Adherence to the sweets-and-treats pattern | | |  |  | Adherence to the health-conscious pattern | | |  |  | Adherence to vegetables-and-processed meats pattern | | |  |
| --- | --- | --- | --- | --- | --- | --- | --- | --- | --- | --- | --- | --- | --- | --- | --- |
|  | All | Low | Moderate | High | *P* |  | Low | Moderate | High | *P* |  | Low | Moderate | High | *P* |
| *n* (%) | 721 (100) | 240 (33) | 241 (33) | 240 (33) |  |  | 240 (33) | 241 (33) | 240 (33) |  |  | 240 (33) | 241 (33) | 240 (33) |  |
| Boys, *n* (%) | 368 (51) | 121 (50) | 129 (54) | 118 (49) | 0.615 |  | 130 (54) | 119 (49) | 119 (50) | 0.494 |  | 127 (53) | 108 (45) | 133 (55) | 0.052 |
| Age, years: |  |  |  |  |  |  |  |  |  |  |  |  |  |  |  |
| Mean (SD) | 4.7 (0.89) | **4.7 (0.84)** | **4.7 (0.89)** | **4.8 (0.94)** | **0.047**^a^ |  | **4.8 (0.92)** | **4.8 (0.88)** | **4.6 (0.87)** | **0.031**^b^ |  | 4.7 (0.90) | 4.7 (0.94) | 4.8 (0.84) | 0.460 |
| Preschool attendance, *n* (%): | |  |  |  |  |  |  |  |  |  |  |  |  |  |  |
| 5 days/week | 454 (63) | **139 (58)** | **166 (69)** | **149 (62)** | **0.043** |  | **175 (73)** | **155 (64)** | **124 (52)** | **<.001** |  | 153 (64) | 163 (68) | 138 (58) | 0.077 |
| Missing | 1 (0.14) | 0 (0) | 0 (0) | 1 (0.42) |  |  | 0 (0) | 0 (0) | 1 (0.41) |  |  | 0 (0) | 0 (0) | 1 (0.42) |  |
| Highest educational level in the family, *n* (%): | | | |  | **0.038** |  |  |  |  | **0.001** |  |  |  |  | 0.108 |
| ≤ Secondary school | 151 (21) | **41 (17)** | **49 (20)** | **61 (25)** |  |  | **64 (27)** | **49 (20)** | **38 (16)** |  |  | 39 (16) | 50 (21) | 62 (26) |  |
| Bachelor’s degree ^c^ | 304 (42) | **102 (43)** | **94 (39)** | **108 (45)** |  |  | **110 (46)** | **99 (41)** | **95 (40)** |  |  | 102 (43) | 102 (42) | 100 (42) |  |
| ≥ Master’s degree | 262 (36) | **96 (40)** | **96 (40)** | **70 (29)** |  |  | **64 (27)** | **93 (39)** | **105 (44)** |  |  | 97 (40) | 88 (37) | 77 (32) |  |
| Missing | 4 (0.55) | 1 (0.42) | 2 (0.83) | 1 (0.42) |  |  | 2 (0.83) | 0 (0) | 2 (0.83) |  |  | 2 (0.83) | 1 (0.42) | 1 (0.42) |  |
| Number of children living in the same household, *n* (%): | | |  |  | 0.124 |  |  |  |  | **0.025** |  |  |  |  | 0.221 |
| 0 | 95 (13) | 29 (12) | 29 (12) | 37 (15) |  |  | **37 (15)** | **23 (9.5)** | **35 (15)** |  |  | 36 (15) | 32 (13) | 27 (11) |  |
| 1 | 400 (55) | 145 (60) | 138 (57) | 117 (49) |  |  | **117 (49)** | **139 (58)** | **144 (60)** |  |  | 139 (58) | 136 (56) | 125 (52) |  |
| ≥ 2 | 226 (31) | 66 (28) | 74 (31) | 86 (36) |  |  | **86 (36)** | **79 (33)** | **61 (25)** |  |  | 65 (27) | 73 (30) | 88 (37) |  |
| Used probiotic supplements, *n* (%): | | |  |  |  |  |  |  |  |  |  |  |  |  |  |
| Yes | 80 (11) | 30 (13) | 26 (11) | 24 (10) | 0.699 |  | **13 (5.4)** | **35 (15)** | **32 (13)** | **0.003** |  | 22 (9.2) | 32 (13) | 26 (11) | 0.349 |
| Missing | 7 (0.97) | 1 (0.42) | 2 (0.83) | 4 (1.7) |  |  | 2 (0.83) | 1 (0.42) | 4 (1.7) |  |  | 3 (1.2) | 3 (1.2) | 1 (0.42) |  |

Age was compared between the dietary pattern thirds using one-way ANOVA, followed by the Tukey HSD post hoc test if significant. Categorical covariates were compared between the dietary pattern thirds using chi-square independence test. Missing values are shown if they were present. Significant differences (*P*<0.05) are indicated in boldface.

**Table S6** (continued)

^a^ No significant differences in the post hoc analysis (*P*>0.05 for each comparison).

^b^ Significant difference between high and moderate adherence in the post hoc analysis (*P*=0.049).

^c^ Or equivalent.

SD, Standard deviation.
